# Cognitive Training for Post-Acute Traumatic Brain Injury: A Systematic Review and Meta-Analysis

**DOI:** 10.3389/fnhum.2016.00537

**Published:** 2016-10-27

**Authors:** Harry Hallock, Daniel Collins, Amit Lampit, Kiran Deol, Jennifer Fleming, Michael Valenzuela

**Affiliations:** ^1^Regenerative Neuroscience Group, Brain and Mind Centre, University of SydneySydney, NSW, Australia; ^2^School of Psychology, University of SydneySydney, NSW, Australia; ^3^Sydney Medical School, University of SydneySydney, NSW, Australia; ^4^School of Health and Rehabilitation Sciences, University of QueenslandBrisbane, QLD, Australia; ^5^Occupational Therapy Department, Princess Alexandra HospitalBrisbane, QLD, Australia; ^6^Centre for Functioning and Health Research, Metro South Hospital and Health ServiceBrisbane, QLD, Australia

**Keywords:** traumatic brain injury, TBI, closed head injury, cognitive training, cognitive outcome, neuropsychological outcome, rehabilitation

## Abstract

**Objective:** To quantitatively aggregate effects of cognitive training (CT) on cognitive and functional outcome measures in patients with traumatic brain injury (TBI) more than 12-months post-injury.

**Design:** We systematically searched six databases for non-randomized and randomized controlled trials of CT in TBI patients at least 12-months post-injury reporting cognitive and/or functional outcomes.

**Main Measures:** Efficacy was measured as standardized mean difference (Hedges’ *g*) of post-training change. We investigated heterogeneity across studies using subgroup analyses and meta-regressions.

**Results:** Fourteen studies encompassing 575 patients were included. The effect of CT on overall cognition was small and statistically significant (*g* = 0.22, 95%CI 0.05 to 0.38; *p* = 0.01), with low heterogeneity (*I*^2^ = 11.71%) and no evidence of publication bias. A moderate effect size was found for overall functional outcomes (*g* = 0.32, 95%CI 0.08 to 0.57, *p* = 0.01) with low heterogeneity (*I*^2^ = 14.27%) and possible publication bias. Statistically significant effects were also found only for executive function (*g* = 0.20, 95%CI 0.02 to 0.39, *p* = 0.03) and verbal memory (*g* = 0.32, 95%CI 0.14 to 0.50, *p* < 0.01).

**Conclusion:** Despite limited studies in this field, this meta-analysis indicates that CT is modestly effective in improving cognitive and functional outcomes in patients with post-acute TBI and should therefore play a more significant role in TBI rehabilitation.

## Introduction

Traumatic brain injury (TBI) causes ongoing disability for millions worldwide (Wilson et al., 2014), with cognitive impairment and psychosocial issues presenting major barriers to positive social outcomes such as community reintegration and employment (Rice-Oxley and Turner-Stokes, 1999). Cognitive impairment in TBI frequently affects the domains of attention, memory, executive functions, processing speed, language, and visuospatial skills (Dikmen et al., 2009). Reviews (Gordon et al., 2006; Cicerone et al., 2011; Lu et al., 2012) have suggested that cognitive rehabilitation for TBI, which encompasses several therapeutic strategies and interventions, can be beneficial for improving these cognitive domains and even community functioning. These interventions may include education, goal-setting, counseling, and internal and external compensation strategies targeting specific cognitive domains.

An on-going issue within the wider field of cognitive rehabilitation is a lack of a consensus for taxonomy of cognitive interventions, including of cognitive training (CT), but here we utilize a working definition consistent across key contributors to the literature (Clare et al., 2003; Buschert et al., 2010; Gates and Valenzuela, 2010). Here, we define and assess the impact of one specific form of cognitive rehabilitation which is seen to be cost-effective, scalable, adaptive (Gates and Valenzuela, 2010): CT. We and others have operationally defined CT to include four main characteristics: (1) repeated practice, (2) on problem-orientated tasks, (3) using standardized stimuli, and (4) targeting specified cognitive domains (Gates and Valenzuela, 2010; Bahar-Fuchs et al., 2013). CT aims to restore impaired skills or harness compensatory mechanisms (Buschert et al., 2010) and can include drill and practice exercises or applied mnemonic strategies. It can be administered either in paper-and-pen format, typically facilitated on a one-on-one basis by a therapist, or computer-assisted CT that can be supervised in a group setting or delivered at home at the individual level. It is therefore important to distinguish CT from the more holistic concept of cognitive rehabilitation that may include aspects of CT targeted to improve cognitive deficits, but also includes non-CT interventions aimed at improving psychological, emotional, motivational, and interpersonal functioning (Gordon et al., 2006).

Restorative treatments and compensatory strategies are generically recommended for the rehabilitation of TBI patients displaying cognitive deficits (INCOG guidelines; (Bayley et al., 2014)). Based on efficacy in other clinical populations (Wykes et al., 2011; Lampit et al., 2014; Leung et al., 2015), CT may have therapeutic potential for TBI. Yet prior reviews of cognitive interventions (Cicerone et al., 2005, 2011; Rees et al., 2007) have not specifically addressed the efficacy of CT for TBI patients. These reviews have attempted to synthesize across mixed samples with various kinds of acquired brain injury (ABI), as well as combine different types of cognitive therapies, and permitted a diversity of study designs. A recent meta-analysis (Rohling et al., 2009) highlights the potential therapeutic benefits of CT for specific brain injury deficits, but similar to the reviews, their study is of mixed etiology and also combines samples of varying time since injury, which although is inevitable, potentially introduces spontaneous recovery as a confounder. However, this could be attenuated by confining research to before or after 12 months post injury.

Accordingly, using a meta-analytic approach, this study aims to systematically evaluate whether operationally defined CT is effective in improving cognitive and functional outcomes at least one-year post-TBI, and to analyze potential moderators that may affect treatment outcomes. The study will analyze individual cognitive and functional domains, as well as overall cognition and overall functioning by pooling the individual domains together, respectively. Investigation of individual domains allows for identification of specific training effects, whilst pooling together individual domains allows for the identification of more general or overall effects that may not be apparent at the individual level for a multitude of reasons such as low sample size or poor study design. Additionally, to investigate potential moderators of training, a sub-group analysis will be conducted. Studies in this field are often small, underpowered and vary in design, thus a meta-analysis can add clarity, as it allows for amalgamation of these small studies to produce an overall analysis with greater statistical power and further reaching conclusions. Thus, as the field of cognitive rehabilitation in TBI is still in its infancy and much more research is required, a meta-analysis could prove crucial in identifying the future direction of CT, and potential design factors that may prove most effective.

## Materials and Methods

This systematic review and meta-analysis adheres to the Preferred Reporting Items for Systematic Reviews and Meta-Analyses (PRISMA) guidelines (Supplementary Table S1), (Liberati et al., 2009) was prospectively registered with PROSPERO (CRD42014013274) and largely follows methods established in our previous meta-analyses (Lampit et al., 2014; Leung et al., 2015).

### Eligibility Criteria

We included both non-randomized and randomized controlled trials (RCTs) provided they investigated the effects of a CT intervention on cognitive and/or functional outcomes in individuals (both intervention and controls groups) with post-acute TBI (time since injury ≥12 months, study mean). Thus we excluded studies that included healthy or acute TBI controls. Eligible outcomes were baseline and post-training performance on measures of cognition, Instrumental Activities of Daily Living (IADL) or dysexecutive functioning, defined as holistic disruptions to frontal lobe functions such as behavior, executive functions and cognition. CT was defined as any intervention incorporating computer-assisted CT, pencil-and-paper-administered CT, or cognitive strategy training, practiced systematically for a minimum of 4 hours. Studies that used combined interventions (e.g., CT with standard physical rehabilitation) were eligible if CT comprised at least 50% of the total intervention duration.

### Search Methodology

We searched CINAHL, the Cochrane Database of Systematic Reviews, EMBASE, MEDLINE, PsycBITE, and PsycINFO databases from inception to July 27, 2015 using a comprehensive search strategy (Supplementary Datasheet 1).

Relevant articles were downloaded to an EndNote library, duplicates were removed and articles from other sources (e.g., references from systematic reviews) were manually added. HH and DC conducted initial screening for eligibility using title and abstract, and then independently examined full-text articles for inclusion.

### Study Appraisal and Risk of Bias Within Studies

A modified form of the Physiotherapy Evidence Database (PEDro) scale (Maher et al., 2003), designed for rating the quality of RCTs, was used by HH and DC to assess the methodological rigor of included studies. As blinding of participants and therapists is impractical in CT trials, these two PEDro items were not assessed, and the maximum overall score (i.e., highest study quality) became 9 (Lampit et al., 2014). Risk of bias resulted from lack of assessor blinding or adherence to intention-to-treat analysis was assessed using the Cochrane’s risk of bias tool (Higgins and Green, 2011). RCTs with high or unclear risk of bias for either of these categories were defined as having a high risk of bias.

### Data Extraction

Cognitive and functional outcome data were extracted in the form of means and standard deviations for each group immediately pre- and post-intervention using a correlation of 0.6 between timepoints or mean group change, and entered into Comprehensive Meta-Analysis version 2 (CMA, Biostat, Englewood, NJ, USA). Coding of outcomes into cognitive domains and effect direction were performed using the *Compendium of Neuropsychological Tests* (Strauss et al., 2006) or by consensus.

### Data Analysis

The dependent variable was standardized mean differences (SMD) (calculated as Hedges’ *g* to correct for small sample sizes) of change from baseline to post-intervention between CT and control groups. Precision of SMD was estimated using 95% confidence intervals (CI). Analyses were conducted on individual cognitive (executive functions, verbal memory, working memory, attention, processing speed, non-verbal memory, visuospatial, language) and functional domains (IADL and dysexecutive functions). Analyses were also conducted on Overall Cognition and Overall Function, which were a result of combining or pooling the respective individual cognitive or functional domains together (Wykes et al., 2011; Lampit et al., 2014). An effect size of *g* < 0.30 was considered small, *g* ≥ 0.30 moderate, and *g* ≥ 0.60 large.

To avoid selective analyses of outcomes, study-level SMDs from the same cognitive domain were combined into a single effect estimate, corrected for inter-correlation across outcomes using a correlation of 0.7 (Gleser and Olkin, 2009). Pooling of outcomes across studies was conducted using random effects model. Heterogeneity across studies was quantified using the *I*^2^ statistic, which quantifies the proportion of variance due to heterogeneity in true effects rather than random error (Higgins et al., 2003). *I*^2^ values of 25, 50, and 75% imply low, moderate, and large heterogeneity, respectively.

To assess publication bias (small-study effect), funnel plots were visually inspected and formally tested using Egger’s Test of the Intercepts if at least 10 studies were available for analysis (Egger et al., 1997; Sterne et al., 2011). If significant asymmetry was detected (*p* < 0.1), we estimated the magnitude of small-study effect using Duval and Tweedie’s Trim and Fill method (Duval and Tweedie, 2000).

In order to detect design factors that may affect CT efficacy, we performed subgroup meta-analyses based on mixed-effects model. Between-subgroup heterogeneity was tested using Cochrane’s Q statistic (significant at *p* < 0.05). Analyses were performed for overall cognitive and overall functional outcomes based on the following study characteristics: study design (randomized or non-randomized), intervention type (combined, strategy or training), control type (active or passive), total hours of training (≤20 or >20 h), session length (≤60 or >60 mins), and session frequency (<4 or ≥4 a week). Univariate meta-regressions were used to detect relationships between cognitive results and PEDro score, sample size and year of publication. All analyses were conducted in CMA.

## Results

### Study Selection

**FIGURE 1:**
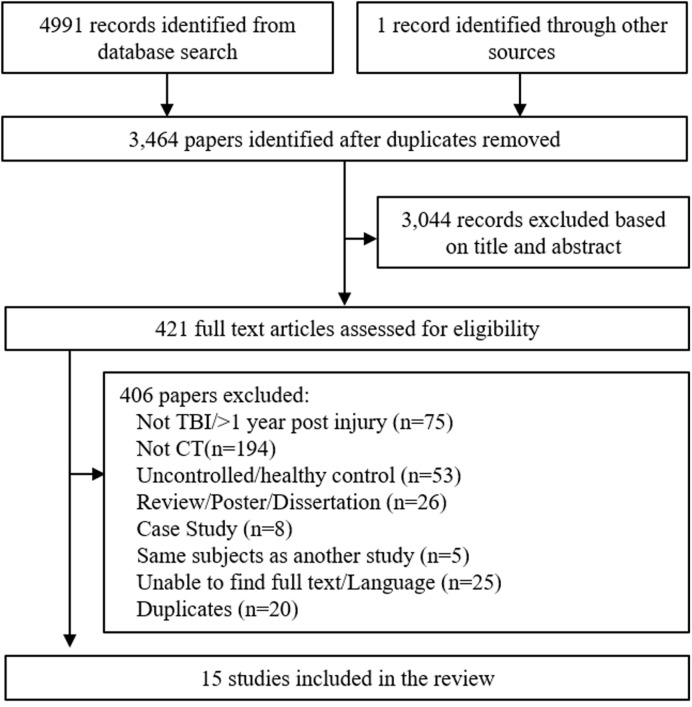
**Flowchart of trial identification and selection.** Note that a single study could be excluded on more than one criterion, but appears only once in the chart.

After removal of duplicates, 3464 articles were screened for inclusion based on published title and abstract. 421 articles were suitable for full-text screening, including one manually added study (**Figure 1**). After full-text screening, 15 studies were eligible for review, however one focused solely on children and adolescence (Thomas-Stonell et al., 1994) and was therefore excluded, leaving 14 studies for analysis. Age was not a screening criterion, but given the fact that TBI can manifest quite differently during adolescent brain development, it was deemed appropriate to exclude this study.

### Characteristics of Included Studies

Data from 575 participants and 169 outcomes were included. The mean number of participants per study was 41.07, with a mean participant age of 38.79 years. Brain injury severity ranged from mild to severe. Time since injury ranged from 1.01 to 14.4 years (*mean* = 5.3 years, *SD* = 3.78). Strategy-based interventions were used in four studies (O’Neil-Pirozzi et al., 2010; Vas et al., 2011; Dawson et al., 2013; Twamley et al., 2014), drill and practice training (including computer-assisted training) in six (Ryan and Ruff, 1988; Baribeau et al., 1989; Ruff et al., 1989; Rattok et al., 1992; Potvin et al., 2011; Nelson et al., 2013), and a combination of both in four studies (Goranson et al., 2003; Tiersky et al., 2005; Wai-Kwong Man et al., 2006; Cantor et al., 2014). An active control group was used in seven studies (**Table 1**). The average PEDro score was 5.64/9 (*SD* = 0.84). Seven studies were RCTs (Ryan and Ruff, 1988; Ruff et al., 1989; Tiersky et al., 2005; Vas et al., 2011; Nelson et al., 2013; Twamley et al., 2014) and seven were non-randomized controlled studies (Baribeau et al., 1989; Rattok et al., 1992; Goranson et al., 2003; Wai-Kwong Man et al., 2006; O’Neil-Pirozzi et al., 2010; Potvin et al., 2011; Dawson et al., 2013; Cantor et al., 2014). Four of the studies confirmed assessor blinding, whilst three reported intention-to-treat.

**Table 1:** Characteristics of included studies.

Author and year	Study design	Mean age (treatment, control)	Injury characteristics	Mean time since injury (treatment, control-years)	Intervention type; total duration (h)	Control type	Outcomes	PEDro score	Risk of bias
Baribeau et al., 1989 (*N* = 41)	Non- randomized controlled trial	27, 27	All severe Closed-head injury	6.58, 5.92	CCT	Active (standard rehab)	Attention	6	N/A
Cantor et al., 2014 (*N* = 91)	Non- RCT	46.7, 43.9	Mixed TBI severity (mild 50%, moderate 19%, severe 31%)	10.7, 14.4	Strategy/CT for executive dysfunction; 90	Passive (wait-list)	Cognitive functioning (multiple domains), IADL, dysexecutive functions	7	N/A
Dawson et al., 2013 (*N* = 13)	Non- RCT	42.6, 40.5	Mixed TBI severity (mild 15%, moderate to severe 85%)	9.8, 10.8	Strategy training for occupational performance; 20	Passive; matched control group	IADL, dysexecutive functions	6	N/A
Goranson et al., 2003 (*N* = 42)	Non-randomized case-control study	34.71, 36.57	Mild to moderate TBI; PTA < 24 h	1.01, 1.12	CT/Strategy training within multidisciplinary rehab; avg. 352	Passive (no rehab)	Community integration	5	N/A
Nelson et al., 2013 (*N* = 36)	RCT	34.8, 31.1	Blast-related mild to moderate TBI; avg. 2.9 blast injuries	2.92, 1.83	CT plus standard rehab; 15	Active (standard rehab)	Cognitive functioning (multiple domains)	6	High
O’Neil-Pirozzi et al., 2010 (*N* = 94)	Non-RCT	47.3, 47	Mixed TBI severity (mild 20%, moderate 22%, severe 47%)	11.8, 13.4	Strategy for memory; 18	Passive (no rehab)	Verbal memory	6	N/A
Potvin et al., 2011 (*N* = 30)	Non-RCT	35.0, 30.9	Mixed TBI severity (moderate 10%, severe 90%)	3.62, 2.83	CT/CCT for prospective memory rehab; 15	Active (education); matched control group	Cognitive functioning (multiple domains), IADL	6	N/A
Rattok et al., 1992^a^ (*N* = 36)	Non-RCT	28.5, 27.1	Severe TBI; days in coma: 36.9 (treatment), 38.9 (control)	3.35, 2.82	CT/CCT within multidisciplinary rehab; 200	Active (multidimensional rehab)	Cognitive functioning (multiple domains)	5	N/A
Ruff et al., 1989 (*N* = 40)	RCT	29.9, 31.7	Moderate to severe TBI; days in coma: 32.1 (treatment), 48.8 (control)	3.18, 4.37	CCT/CT within structured cognitive rehab; 160	Active (non-structured rehab)	Cognitive functioning (multiple domains)	5	High
Ryan and Ruff, 1988 (*N* = 20)	RCT	34.3, 31.4	Head injury with mild to moderate neuropsychological impairment; GCS ≤8 at time of injury	4.54, 4.78	CT/CCT within structured memory rehab; 132	Active (psychosocial rehab)	Cognitive functioning (multiple domains)	4	High
Tiersky et al., 2005 (*N* = 20)	RCT	47.55, 46	Mixed TBI severity (mild 90%)	5.01, 5.47	CT/CBT	Passive (wait-list)	Cognitive functioning (multiple domains), IADL	7	High
Twamley et al., 2014 (*N* = 34)	RCT	29.4, 34.3	Mild to moderate TBI; loss of consciousness <24 hrs	3.6, 5.1	Strategy training within supported employment program; 12	Active (supported employment)	Cognitive functioning (multiple domains), daily functioning,	5	High
Vas et al., 2011 (*N* = 28)	RCT	39.0, 47.0	Mild to severe TBI; PTA: 2.1 & 2.46 weeks; moderate functional impairment.	1.39, 1.36	Strategy training for higher-order reasoning; 18	Active (psychoeducation)	Cognitive functioning (multiple domains), IADL	6	High
Wai-Kwong Man et al., 2006 (*N* = 50)	Non-RCT	44.87, 48.55	Not specified	3.48, 4.13	CCT/Strategy training for problem-solving; 20 sessions	Passive (no treatment); matched control group	Executive function, IADL	5	N/A

*RCT, randomized controlled trial; TBI, traumatic brain injury; IADL, Instrumental Activities of Daily Living; CT, cognitive training; CCT, computerized cognitive training; GCS, Glasgow coma scale; PTA, Post-traumatic amnesia. ^a^Three treatment groups - 1 group excluded, 95% TBI and 5% cerebral anoxia.*

### Efficacy on Overall Cognitive Outcomes

**FIGURE 2:**
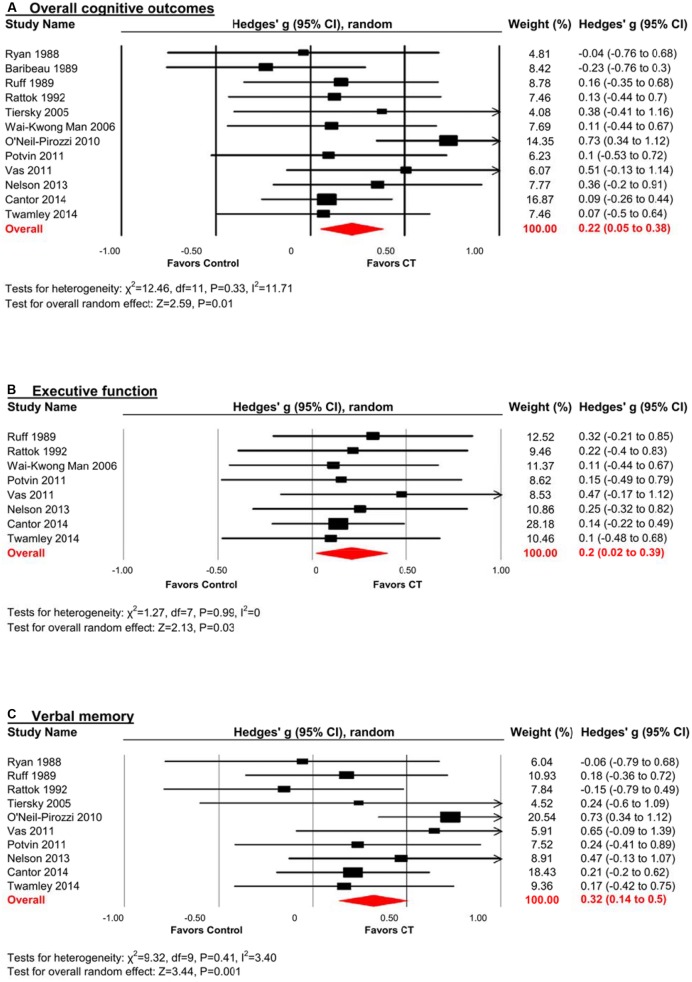
**Efficacy of cognitive training (CT) on (A) overall cognitive outcomes; (B) executive function; and (C) verbal memory.** Effect estimates are based on a random-effects model.

**FIGURE 3:**
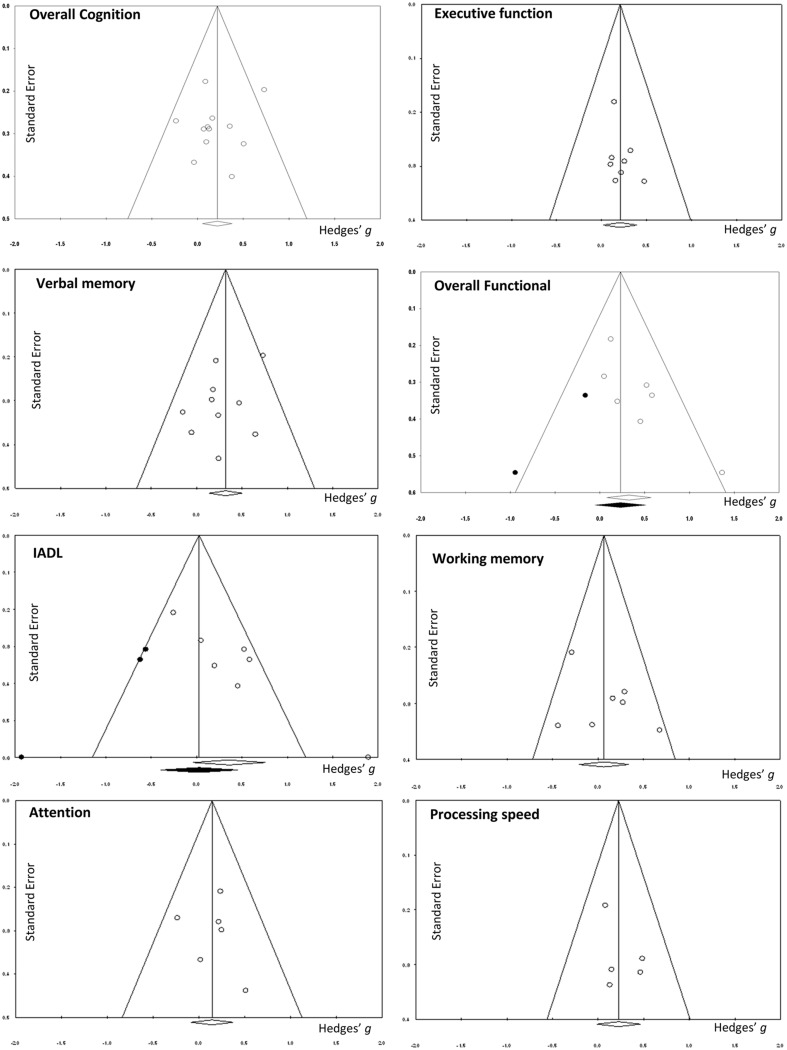
**Funnel plots**.

There was a small, statistically significant positive effect of CT on overall cognitive outcomes (*k* = 12, *g* = 0.22, 95% CI 0.05 to 0.38, *p* = 0.01; **Figure 2A**). Heterogeneity across studies was low (*I*^2^ = 11.71%, 95% CI 0% to 51.39%). A funnel plot of results did not reveal asymmetry (Egger’s intercept = -0.93, *p* = 0.51; **Figure 3**) suggesting no significant evidence of systematic bias toward including positive (or negative) outcomes.

### Efficacy on Specific Cognitive Domains

#### Executive Function

The effect size was small and statistically significant (*k* = 8, *g* = 0.20, 95% CI 0.02 to 0.39, *p* = 0.03; **Figure 2B**). Statistical heterogeneity across studies was zero (*I*^2^ = 0, 95% CI 0% to 0%), and the funnel plot did not show evidence of asymmetry (**Figure 3**).

#### Verbal Memory

The effect size was moderate and statistically significant (*k* = 10, *g* = 0.32, 95% CI 0.14 to 0.50, *p* < 0.01; **Figure 2C**). Statistical heterogeneity across studies was small (*I*^2^ = 3.40, 95% CI 0% to 63.84%), and the funnel plot did not show evidence of asymmetry (Egger’s intercept = -1.69, *p* = 0.21; **Figure 3**).

#### Working Memory

**FIGURE 4:**
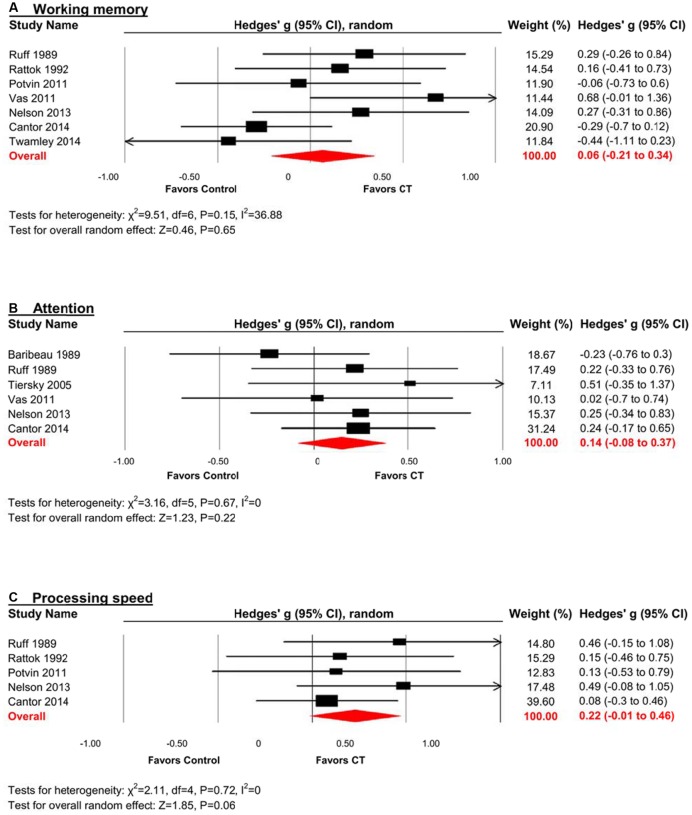
**Efficacy of CT on (A) working memory; (B) attention; and (C) processing speed.** Effect estimates are based on a random-effects model.

The effect size was small and statistically non-significant (*k* = 7, *g* = 0.06, 95% CI -0.21 to 0.34, *p* = 0.94; **Figure 4A**). Statistical heterogeneity across studies was moderate (*I*^2^ = 36.88, 95% CI 0% to 73.44%), and the funnel plot did not show evidence of asymmetry (**Figure 3**).

#### Attention

The effect size was small and statistically non-significant (*k* = 6, *g* = 0.14, 95% CI -0.09 to 0.37, *p* = 0.22; **Figure 4B**). Statistical heterogeneity across studies was zero (*I*^2^ = 0, 95% CI 0% to 61.04%), and the funnel plot did not show evidence of asymmetry (**Figure 3**).

#### Processing Speed

The effect size was small and statistically non-significant (*k* = 5, *g* = 0.22, 95% CI -0.01 to 0.46, *p* = 0.06; **Figure 4C**). Statistical heterogeneity across studies was zero (*I*^2^ = 0, 95% CI 0% to 62.91%), and the funnel plot did not show evidence of asymmetry (**Figure 3**).

#### Non-verbal Memory

**FIGURE 5:**
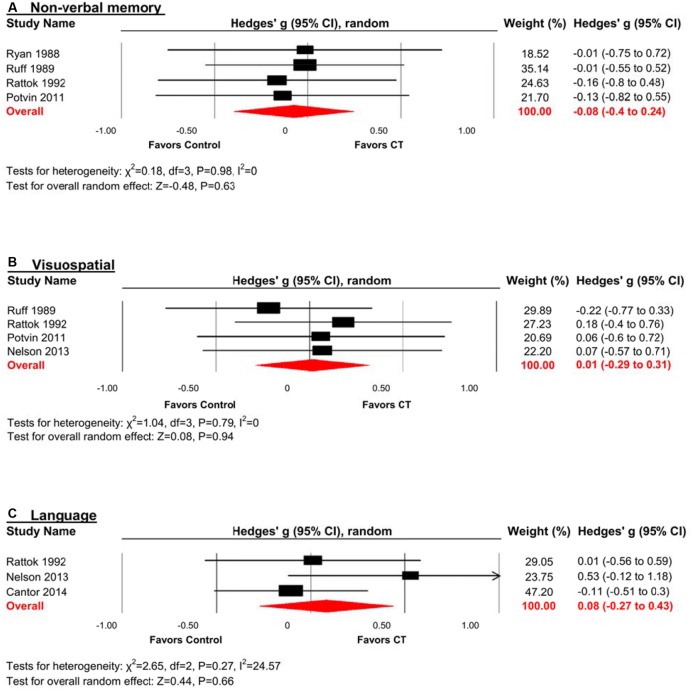
**Efficacy of CT on (A) non-verbal memory; (B) visuospatial; and (C) language.** Effect estimates are based on a random-effects model.

The effect size was negative, small and statistically non-significant (*k* = 4, *g* = -0.08, 95% CI -0.40 to 0.24, *p* = 0.63; **Figure 5A**). Statistical heterogeneity across studies was zero (*I*^2^ = 0, 95% CI 0% to 0%), and the funnel plot did not show evidence of asymmetry.

#### Visuospatial

The effect size was small and statistically non-significant (*k* = 4, *g* = 0.01, 95% CI -0.29 to 0.31, *p* = 0.94; **Figure 5B**). Statistical heterogeneity across studies was zero (*I*^2^ = 0, 95% CI 0% to 62.81%), and the funnel plot did not show evidence of asymmetry.

#### Language

The effect size was small and statistically non-significant (*k* = 3, *g* = 0.08, 95% CI -0.27 to 0.43, *p* = 0.66; **Figure 5C**). Statistical heterogeneity across studies was small (*I*^2^ = 24.57, 95% CI 0% to 97.47%), and the funnel plot did not show evidence of asymmetry.

### Efficacy on Overall Functional Outcomes

**FIGURE 6:**
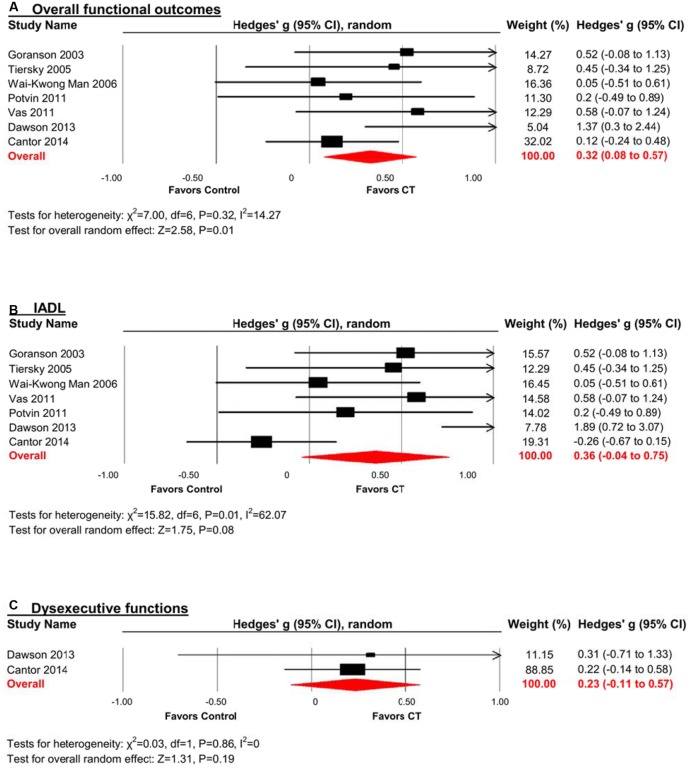
**Efficacy of CT on (A) overall functional outcomes; (B) instrumental activities of daily living (IADL); and (C) dysexecutive functions.** Effect estimates are based on a random-effects model.

A pooled analysis of the seven studies reporting functional outcomes revealed a moderate and statistically significant effect size (*g* = 0.32, 95% CI 0.08 to 0.57, *p* = 0.01; **Figure 6A**). Heterogeneity across studies was low (*I*^2^ = 14.27%, 95% CI 0% to 75.39%). The funnel plot revealed asymmetry, indicating more positive results in smaller studies. A trim and fill analysis revealed a smaller and statistically non-significant effect size (*g* = 0.23, 95% CI -0.05 to 0.51, *p* = 0.11 **Figure 3**).

### Efficacy on Specific Functional Domains

#### Instrumental Activities of Daily Living (IADL)

The effect size was moderate and statistically non-significant (*k* = 7, *g* = 0.36, 95% CI -0.04 to 0.75, *p* = 0.08; **Figure 6B**). Statistical heterogeneity across studies was moderate (*I*^2^ = 62.07%, 95% CI 13.59% to 83.35%). The funnel plot showed evidence of asymmetry, but trim and fill analysis did not alter the effect size (**Figure 3**).

#### Dysexecutive Functions

The effect size was small and statistically non-significant (*k* = 2, *g* = 0.23, 95% CI -0.11 to 0.57, *p* = 0.19; **Figure 6C**). Statistical heterogeneity across studies was zero (*I*^2^ = 0).

### Moderators of CT Efficacy

**FIGURE 7:**
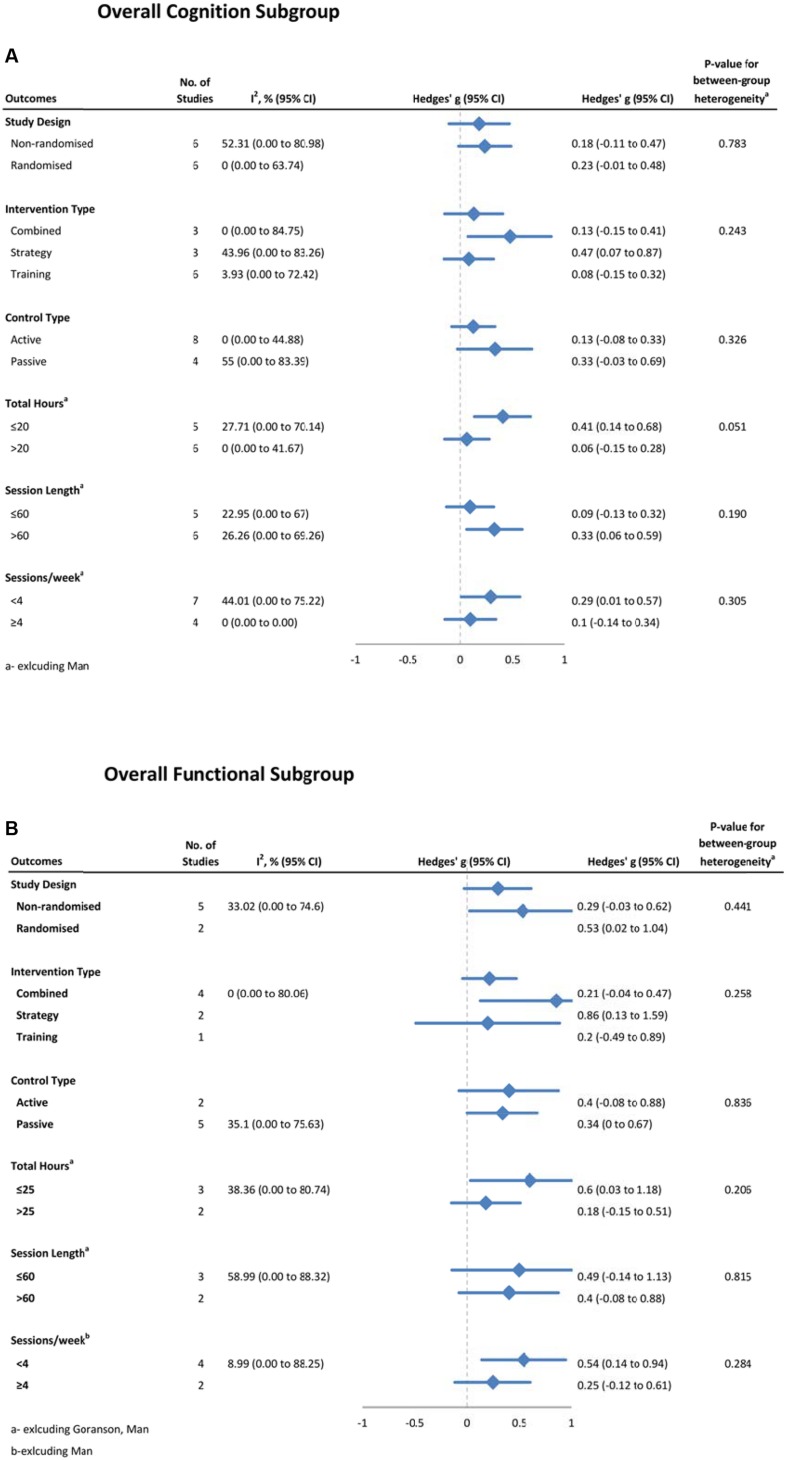
**Subgroup analysis of moderators for (A) overall cognitive outcomes, and (B) overall functional outcomes**.

Possible moderators of training effects on overall cognitive (**Figure 7A**) and functional (**Figure 7B**) outcomes were investigated using sub-group analyses. For overall cognition, we did not find significant between group differences for study design, intervention type, control type, total hours of training, session length or session frequency. However there was a strong trend toward less training being more effective on overall cognition, with studies providing 20 h or less of training (*g* = 0.41, 95% CI 0.14 to 0.68, *p* < 0.01, *I*^2^ = 21.40%) being more effective than those that provided more than 20 hours (*g* = 0.06, 95% CI -0.15 to 0.28, *p* = 0.55; *Q* = 3.80, df = 1, *p* = 0.05). To further investigate this trend, we conducted an analysis on a *post hoc* basis. This correlation comparing length of training and severity of injury was found to be non-significant (*r* = 0.26, *p* = 0.44, *n* = 11). There were no significant between-subgroup differences with overall functional outcomes for any of these moderators. As both IADL and working memory outcomes had moderate heterogeneity, subgroup analyses were conducted, but no significant differences were found for either. For other domains, heterogeneity was close to zero, thus subgroup analyses were not warranted.

Meta-regression showed no statistically significant relationships between overall cognitive effects and PEDro score (β = 0.06, *p* = 0.57), sample size (β = 0.004, *p* = 0.23), or year of publication (β = 0.01, *p* = 0.18).

**FIGURE 8:**
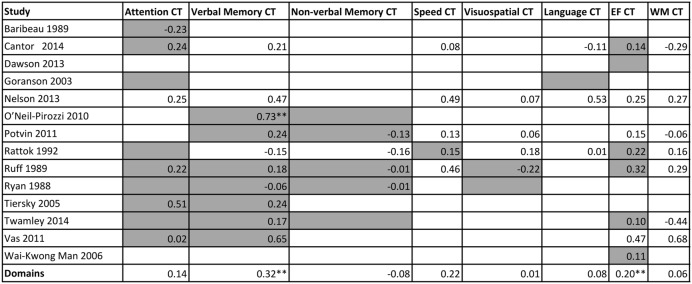
**Matrix of training content against effect size of each cognitive outcome from the individual studies**. Gray cells indicate the study trained in the domain. EF = executive functions; WM = working memory. ^∗∗^*p* < 0.05

A matrix was constructed to investigate whether the content of training (the domain/s that were trained) moderated outcomes on specific cognitive domains outcomes, i.e., if there was transfer. A summary of these cognitive outcomes is presented in **Figure 8**, and categorized by study and cognitive domain trained. No statistical analysis was run on this data, but the matrix illustrates which cognitive domains were trained (gray color cells), and the effect sizes at a study level or pooled together at a domain level.

## Discussion

Cognitive-based interventions are effective in several clinical populations (Wykes et al., 2011; Lampit et al., 2014; Leung et al., 2015), and here we expand the evidence base to include post-acute TBI. CT was particularly effective on overall cognition, as well as the cognitive domains of verbal memory and executive function, and jointly improved individuals’ IADLs whilst reducing severity of dysexecutive signs and symptoms.

TBI is extremely heterogeneous in its etiology and origins. Accordingly, patients present with a variety of cognitive deficits (Dikmen et al., 2009), with information processing speed and verbal memory most commonly affected (Skandsen et al., 2010). It is therefore promising that this study found not only general cognitive efficacy, but specific efficacy for executive function and verbal memory. Contrary to this, a previous meta-analysis (Rohling et al., 2009) found that cognitive rehabilitation was not effective in TBI patients. However, that study combined several different types of cognitive interventions and patients varied greatly in the time since injury. As mentioned, cognitive rehabilitation encompasses a variety of therapeutic approaches, and here we aim to focus on CT as operationally defined in the introduction. Moreover, timing of intervention may well be critical. TBI often progresses through stages of unconsciousness and emerging consciousness; confusion with dense anterograde amnesia that can vary from days to several weeks; and a long-term period of restoration of cognitive, neuropsychological and social functioning that can last for several years (Povlishock and Katz, 2005). Here we have clarified the literature to some extent and shown that one approach to cognitive rehabilitation, CT, is effective for certain cognitive domains in the post-acute phase.

Cognitive rehabilitation, which can include CT, is known to improve community functioning even several years after TBI (Gordon et al., 2006). Our analyses suggest that CT may itself be sufficient to retrain functional skills or facilitate compensatory mechanisms that can translate into everyday outcomes. In the TBI literature, functionality is often measured by IADL scales and assessment of dysexecutive syndrome. Given the importance of both IADLs and dysexecutive syndrome to everyday life, it is noteworthy that CT produced a moderate effect size on these outcomes when combined. Furthermore, the low heterogeneity surrounding this estimate indicates that the result is subject to little explainable variation and is thus an accurate estimate of effect size. Whilst the combination of these two outcomes may appear to be novel, previous studies have shown loose connections between the two (Pa et al., 2009; Marshall et al., 2011). Importantly, this result suggests that CT has the potential to achieve so-called “far-transfer” (Barnett and Ceci, 2002) to positively influence real world issues faced by TBI patients.

Despite these combined results, IADL or dysexecutive functioning did not produce significant improvements when considered separately. This may be due to insufficient power, as not only were there limited studies examining these outcomes, and small sample sizes, but a separate analysis of the two domains displayed larger CI. Positive effects on daily function were restricted to a pooled analysis of combined dysexecutive and IADL outcomes. Whilst this approach has some precedence (Pa et al., 2009; Marshall et al., 2011) and was planned a priori, when each type of outcome was considered individually no significant effects were observed. This therefore brings up the issue as to what can be reasonably combined in terms of outcomes measures within a meta-analysis – a topic treated in detail by Borenstein et al. (2009). In their example, combining tests of Maths and English is justified, “If our goal is to assess the impact on performance in general, then the answer is Yes.” (Borenstein et al., 2009, p. 357). Our goal was to assess the impact of CT on those areas that most impact day to day function in TBI rehabilitation, inclusive of both dysexecutive syndrome (Rao and Lyketsos, 2000) and impaired IADLs (Colantonio et al., 2004). Hence, there are promising indications that CT can help support daily function in chronic TBI patients, but clearly more research is required to parse these effects out.

Interestingly, CT had a significant effect on executive function but not dysexecutive outcomes. This may appear paradoxical given the two outcomes are intrinsically (and inversely) related (Ardila, 2013). However, this pattern of results can be explained by the nature of the data. Executive outcomes originate from neuropsychological tests that are generally objective, quantitative and continuous, and thus sensitive to change, whilst dysexecutive instruments are generally subjective, qualitative and ordinal. By nature these instruments are therefore of lower resolution and require much larger behavioral change before detection. Further research is therefore required to determine whether CT can improve not just psychometric executive function but also minimize the presentation or severity of dysexecutive symptoms in post-acute TBI.

Of the potential moderators analyzed, a strong trend was found only for training hours. Studies where subjects trained for ≤ 20 h showed improvements in overall cognition compared to studies where patients trained more. This is consistent with evidence of weaker effect sizes in studies that provided intense training schedules (Lampit et al., 2014) or long training durations (Toril et al., 2014) in healthy older adults. A possible explanation for this trend could be the heterogeneity in injury severity amongst the population, whereby those with more severe injuries required more training, with the assumption that increased severity means lower improvement. We conducted a *post hoc* analysis to test this theory, but we did not find a relationship between length of training and injury severity across studies. However given that this *post hoc* analysis was conducted on such a small sample size, and thus lacks power, we cannot completely rule out that the trend in training time is linked with injury severity. Nonetheless, it is intriguing that across different clinical cohorts there may be converging evidence for the importance of avoiding over-dosing, or over-training participants. This concept is even more salient in the field of TBI, where rehabilitation is often guided by the principle that greater intensity or number of repetitions is better. Here, we conclude that CT at a circumscribed dose, at the right time in the post-acute stage, is preferable.

Other possible moderators analyzed were found to be non-significant, consistent with the small number of studies and minimal explainable between-study variance – a concept we have previously discussed (Leung et al., 2015). More specifically, our data suggests that an important study design factor, whether randomization occurred or not, did not impact CT efficacy in post-acute TBI. However, we cannot rule out that this could be due to a lack of power, a notion counter-weighed by similar effect sizes from the two design methods. This finding supports our decision to combine both non randomized and RCTs into a single analysis.

To further explore moderating or driving factors of cognitive outcomes, we investigated whether there was a link between training content and cognitive outcomes (**Figure 8**). For this population, cross-transfer, the idea that training in one domain can result in improvements in another untrained domain, appears to be unlikely. This is evident when looking at the columns for working memory, speed, language and executive functions. We can see here, as indicated by the gray cells, or lack thereof, that there was minimal training on these domains, however there was training in many other domains. The fact that there are no significant results, in addition to the obvious lack of power, suggests a lack of cross transfer, a sentiment mirrored in previous research (Edwards et al., 2002). Importantly, we cannot conclude that certain domains, such as working memory, speed, language, and executive functions are ineffective or non-responsive in this population. Instead, this figure suggests that there is need for more trials that are training or targeting multiple different cognitive domains.

Limitations include potential selection bias that may have influenced results. Our narrow eligibility criteria and decision to include only studies published in English, resulted in well-controlled CT studies being excluded from this analysis, such as trials implemented before 12 months post-injury. We chose this temporal window for clinical reasons, namely to minimize the confounding effects of spontaneous recovery of function that can occur during the acute and sub-acute stages (Sohlberg and Mateer, 2001). A caveat to this criterion was a reliance on study-level characteristic. Some studies included participants from 3 months post-injury and onward, resulting in large variations in time since injury, despite the reported study average being >12 months post injury. To further clarify the specificity of our findings to this temporal window, a patient-level meta-analysis is required. In addition, our decision to only include functional outcomes that could be categorized as IADL or dysexecutive functioning was a potential source of selection bias, but a decision we consider clinically principled since functional outcomes from three studies e.g., ‘Life-3’ (Dawson et al., 2013; Cantor et al., 2014; Twamley et al., 2014) were idiosyncratic and deemed incomparable.

A notable limitation of our analysis is the heterogeneity in injury severity, but this is reflective of the state of the field. Indeed, many of the included studies themselves comprised patients of varying TBI severity (from mild to severe). However, low statistical heterogeneity indicates that there was no other important source of bias between study variance besides total hours of training. Average PEDro quality scores were relatively low, but this was mainly attributable to two points being allocated for randomization procedures. We specifically tested this factor and found it was not influencing effect size estimates. Perhaps the largest limitation of our study is the relative infancy of the field. With only six of the studies included being RCTs, the field is somewhat nascent, thus our results must be viewed with some skepticism. Nonetheless there is enough power to show effectiveness of CT on overall cognitive and functional outcomes, however, clearly future research with more rigorous trial design and reporting is required.

TBI is fundamentally heterogeneous and manifests in complex and unpredictable patterns, resulting in diverse physical, behavioral, cognitive and functional outcomes. Discerning therapeutic efficacy in this population is therefore challenging. Despite this potential for background ‘noise’ and the limited studies in a still developing field, we found encouraging results with implications for clinical practice. Namely, significant cognitive gains were seen as a result of CT more than one-year post-injury when spontaneous neurological recovery is assumed to have stabilized. It is encouraging to see that there may be a possible link between training intensity and overall efficacy, but further studies with larger sample sizes and more heterogeneous populations are required to explore this relationship. Small samples and lack of power have meant that the effectiveness of CT in TBI has been inconclusive in individual studies – this is precisely the condition when a meta-analysis can add value and clarity to a field (Borenstein et al., 2009). This meta-analysis thereby provides evidence that CT may be modestly effective in promoting cognitive and functional gains in everyday life. Accordingly, further investigation of different approaches to CT is required along with health economic analyses of the costs and benefits of CT for post-acute TBI.

## Author Contributions

HH, DC, KD and MV: Design and/or conceptualization of the study. HH, DC, JF, AL and MV: Analysis and/or interpretation of the data. HH, DC, KD, JF, AL and MV: Drafting and/or revising the manuscript.

## Conflict of Interest Statement

MV and AL receive in-kind research support in the form of no-cost software from BrainTrain Inc. and Synaptikon GmbH for projects unrelated to this work. All the other authors declare that the research was conducted in the absence of any commercial or financial relationships that could be construed as a potential conflict of interest. The reviewer JR and handling Editor declared their shared affiliation, and the handling Editor states that the process nevertheless met the standards of a fair and objective review.

## References

[B1] Ardila A. (2013). There are two different dysexecutive syndromes. *J. Neurol. Disord.* 1 1–4. 10.4172/2329-6895.1000114

[B2] Bahar-Fuchs A. Clare L. Woods B. (2013). Cognitive training and cognitive rehabilitation for mild to moderate Alzheimer’s disease and vascular dementia. *Cochrane Database Syst. Rev.* 6 CD003260. 10.1002/14651858.CD003260.pub2 PMC7144738 23740535

[B3] Baribeau J. Ethier M. Braun C. (1989). A neurophysiological assessment of selective attention before and after cognitive remediation in patients with severe closed head injury. *J. Neurol. Rehabil.* 3 71–92.

[B4] Barnett S. M. Ceci S. J. (2002). When and where do we apply what we learn? A taxonomy for far transfer. *Psychol. Bull.* 128 612–637. 10.1037/0033-2909.128.4.612 12081085

[B5] Bayley M. T. Tate R. Douglas J. M. Turkstra L. S. Ponsford J. Stergiou-Kita M. (2014). Incog guidelines for cognitive rehabilitation following traumatic brain injury: methods and overview. *J. Head Trauma Rehabil.* 29 290–306. 10.1097/HTR.0000000000000070 24984093

[B6] Borenstein M. Hedges L. Higgins J. P. Rothstein H. R. (2009). *Introduction to Meta-Analysis.* Chichester: Wiley.

[B7] Buschert V. Bokde A. L. Hampel H. (2010). Cognitive intervention in Alzheimer disease. *Nat. Rev. Neurol.* 6 508–517. 10.1038/nrneurol.2010.113 20717104

[B8] Cantor J. Ashman T. Dams-O’connor K. Dijkers M. P. Gordon W. Spielman L. (2014). Evaluation of the short-term executive plus intervention for executive dysfunction after traumatic brain injury: a randomized controlled trial with minimization. *Arch. Phys. Med. Rehabil.* 95 1e–9.e. 10.1016/j.apmr.2013.08.005 23988395

[B9] Cicerone K. D. Dahlberg C. Malec J. F. Langenbahn D. M. Felicetti T. Kneipp S. (2005). Evidence-based cognitive rehabilitation: updated review of the literature from 1998 through 2002. *Arch. Phys. Med. Rehabil.* 86 1681–1692. 10.1016/j.apmr.2005.03.024 16084827

[B10] Cicerone K. D. Langenbahn D. M. Braden C. Malec J. F. Kalmar K. Fraas M. (2011). Evidence-based cognitive rehabilitation: updated review of the literature from 2003 through 2008. *Arch. Phys. Med. Rehabil.* 92 519–530. 10.1016/j.apmr.2010.11.015 21440699

[B11] Clare L. Woods R. T. Moniz Cook E. D. Orrell M. Spector A. (2003). Cognitive rehabilitation and cognitive training for early-stage Alzheimer’s disease and vascular dementia. *Cochrane Database Syst. Rev.* 4:CD003260. 10.1002/14651858.CD003260 14583963

[B12] Colantonio A. Ratcliff G. Chase S. Kelsey S. Escobar M. Vernich L. (2004). Long-term outcomes after moderate to severe traumatic brain injury. *Disabil. Rehabil.* 26 253–261. 10.1080/09638280310001639722 15200240

[B13] Dawson D. R. Binns M. A. Hunt A. Lemsky C. Polatajko H. J. (2013). Occupation-based strategy training for adults with traumatic brain injury: a pilot study. *Arch. Phys. Med. Rehabil.* 94 1959–1963. 10.1016/j.apmr.2013.05.021 23796683

[B14] Dikmen S. S. Corrigan J. D. Levin H. S. Machamer J. Stiers W. Weisskopf M. G. (2009). Cognitive outcome following traumatic brain injury. *J. Head Trauma Rehabil.* 24 430–438. 10.1097/HTR.0b013e3181c133e9 19940676

[B15] Duval S. Tweedie R. (2000). Trim and fill: a simple funnel-plot-based method of testing and adjusting for publication bias in meta-analysis. *Biometrics* 56 455–463. 10.1111/j.0006-341X.2000.00455.x 10877304

[B16] Edwards J. Wadley V. Myers R. Roenker D. L. Cissell G. Ball K. (2002). Transfer of a speed of processing intervention to near and far cognitive functions. *Gerontology* 48 329–340. 10.1159/000065259 12169801

[B17] Egger M. Davey Smith G. Schneider M. Minder C. (1997). Bias in meta-analysis detected by a simple, graphical test. *BMJ* 315:629 10.1136/bmj.315.7109.629 PMC2127453 9310563

[B18] Gates N. Valenzuela M. J. (2010). Cognitive exercise and its role in cogitive function in older adults. *Curr. Psychiatry Rep.* 12 20–27. 10.1007/s11920-009-0085-y 20425306

[B19] Gleser L. J. Olkin I. (2009). “Stochastically dependent effect sizes,” in *The Handbook of Research Synthesis and Meta-Analysis*, 2nd Edn, eds Cooper H. Hedges L. Valentine J. (New York, NY: Russell Sage Foundation).

[B20] Goranson T. E. Graves R. E. Allison D. La Freniere R. (2003). Community integration following multidisciplinary rehabilitation for traumatic brain injury. *Brain Injury* 17 759–774. 10.1080/0269905031000088513 12850942

[B21] Gordon W. A. Zafonte R. Cicerone K. Cantor J. Brown M. Lombard L. (2006). Traumatic brain injury rehabilitation: state of the science. *Am. J. Phys. Med. Rehabil.* 85 343–382. 10.1097/01.phm.0000202106.01654.61 16554685

[B22] Higgins J. Green S. (eds) (2011). *Cochrane Handbook for Systematic Reviews of Interventions Version 5.1.0: The Cochrane Collaboration*. Available at: http://www.cochrane-handbook.org [accessed Nov 14, 2011]

[B23] Higgins J. P. Thompson S. G. Deeks J. J. Altman D. G. (2003). Measuring inconsistency in meta-analyses. *BMJ* 327:557 10.1136/bmj.327.7414.557 PMC192859 12958120

[B24] Lampit A. Hallock H. Valenzuela M. (2014). Computerized cognitive training in cognitively healthy older adults: a systematic review and meta-analysis of effect modifiers. *PLoS Med.* 11:e1001756 10.1371/journal.pmed.1001756 PMC4236015 25405755

[B25] Leung I. H. K. Walton C. C. Hallock H. Lewis S. J. G. Valenzuela M. Lampit A. (2015). Cognitive training in Parkinson disease: a systematic review and meta-analysis. *Neurology* 85 1843–1851. 10.1212/WNL.0000000000002145 26519540 PMC4662707

[B26] Liberati A. Altman D. G. Tetzlaff J. Mulrow C. Gotzsche P. C. Ioannidis J. P. (2009). The Prisma statement for reporting systematic reviews and meta-analyses of studies that evaluate healthcare interventions: explanation and elaboration. *BMJ* 339:b2700 10.1136/bmj.b2700 PMC2714672 19622552

[B27] Lu J. Gary K. W. Neimeier J. P. Ward J. Lapane K. L. (2012). Randomized controlled trials in adult traumatic brain injury. *Brain Inj.* 26 1523–1548. 10.3109/02699052.2012.722257 23163248

[B28] Maher C. G. Sherrington C. Herbert R. D. Moseley A. M. Elkins M. (2003). Reliability of the Pedro scale for rating quality of randomized controlled trials. *Phys. Ther.* 83 713–721. 12882612

[B29] Marshall G. A. Rentz D. M. Frey M. T. Locascio J. J. Johnson K. A. Sperling R. A. (2011). Executive function and instrumental activities of daily living in MCI and AD. *Alzheimers Dement.* 7 300–308. 10.1016/j.jalz.2010.04.005 21575871 PMC3096844

[B30] Nelson L. A. Macdonald M. Stall C. Pazdan R. (2013). Effects of interactive metronome therapy on cognitive functioning after blast-related brain injury: a randomized controlled pilot trial. *Neuropsychology* 27 666–679. 10.1037/a0034117 24059443

[B31] O’Neil-Pirozzi T. M. Strangman G. E. Goldstein R. Katz D. I. Savage C. R. Kelkar K. (2010). A controlled treatment study of internal memory strategies (I-Mems) following traumatic brain injury. *J. Head Trauma Rehabil.* 25 43–51. 10.1097/HTR.0b013e3181bf24b1 20051897

[B32] Pa J. Boxer A. Chao L. L. Gazzaley A. Freeman K. Kramer J. (2009). Clinical-neuroimaging characteristics of dysexecutive mild cognitive impairment. *Ann. Neurol.* 65 414–423. 10.1002/ana.21591 19399879 PMC2680500

[B33] Potvin M. J. Rouleau I. Senechal G. Giguere J. F. (2011). Prospective memory rehabilitation based on visual imagery techniques. *Neuropsychol. Rehabil.* 21 899–924. 10.1080/09602011.2011.630882 22150454

[B34] Povlishock J. T. Katz D. I. (2005). Update of neuropathology and neurological recovery after traumatic brain injury. *J. Head Trauma Rehabil.* 20 76–94. 10.1097/00001199-200501000-00008 15668572

[B35] Rao V. Lyketsos C. (2000). Neuropsychiatric sequelae of traumatic brain injury. *Psychosomatics* 41 95–103. 10.1176/appi.psy.41.2.95 10749946

[B36] Rattok J. Ross B. Ben-Yishay Y. Ezrachi O. Silver S. Lakin P. (1992). Outcome of different treatment mixes in a multidimensional neuropsychological rehabilitation program. *Neuropsychology* 6 395–415. 10.1037/0894-4105.6.4.395

[B37] Rees L. Marshall S. Hartridge C. Mackie D. Weiser M. (2007). Cognitive interventions post acquired brain injury. *Brain Inj.* 21 161–200. 10.1080/02699050701201813 17364530

[B38] Rice-Oxley M. Turner-Stokes L. (1999). Effectiveness of brain injury rehabilitation. *Clin. Rehabil.* 13(Suppl. 1), 7–24. 10.1191/026921599668051623 10685619

[B39] Rohling M. L. Faust M. E. Beverly B. Demakis G. (2009). Effectiveness of cognitive rehabilitation following acquired brain injury: a meta-analytic re-examination of Cicerone et al.’s (2000, 2005) systematic reviews. *Neuropsychology* 23 20–39. 10.1037/a0013659 19210030

[B40] Ruff R. M. Baser C. A. Johnston J. W. Marshall L. F. Klauber S. K. Klauber M. R. (1989). Neuropsychological rehabilitation: an experimental study with head-injured patients. *J. Head Trauma Rehabil.* 4 20–36. 10.1097/00001199-198909000-00006

[B41] Ryan T. V. Ruff R. M. (1988). The efficacy of structured memory retraining in a group comparison of head trauma patients. *Arch. Clin. Neuropsychol.* 3 165–179. 10.1016/0887-6177(88)90061-3 14591268

[B42] Skandsen T. Finnanger T. G. Andersson S. Lydersen S. Brunner J. F. Vik A. (2010). Cognitive impairment 3 months after moderate, and severe traumatic brain injury: a prospective follow-up study. *Arch. Phys. Med. Rehabil.* 91 1904–1913. 10.1016/j.apmr.2010.08.021 21112433

[B43] Sohlberg M. M. Mateer C. A. (2001). *Cognitive Rehabilitation: An Integrative Neuropsychological Approach*. New York, NY: The Guilford Press.

[B44] Sterne J. A. Sutton A. J. Ioannidis J. P. Terrin N. Jones D. R. Lau J. (2011). Recommendations for examining and interpreting funnel plot asymmetry in meta-analyses of randomised controlled trials. *BMJ* 343:d4002 10.1136/bmj.d4002 21784880

[B45] Strauss S. Sherman E. Spreen O. (2006). *A compendium of Neuropsychological Tests: Administration, Norms and Commentary*. Oxford: Oxford University Press.

[B46] Thomas-Stonell N. Johnson P. Schuller R. Jutai J. (1994). Evaluation of a computer-based program for remediation of cognitive-communication skills. *J. Head Trauma Rehabil.* 9 25–37. 10.1097/00001199-199412000-00005

[B47] Tiersky L. A. Anselmi V. Johnston M. V. Kurtyka J. Roosen E. Schwartz T. (2005). A trial of neuropsychologic rehabilitation in mild-spectrum traumatic brain injury. *Arch. Phys. Med. Rehabil.* 86 1565–1574. 10.1016/j.apmr.2005.03.013 16084809

[B48] Toril P. Reales J. M. Ballesteros S. (2014). Video game training enhances cognition of older adults: a meta-analytic study. *Psychol. Aging* 29 706–716. 10.1037/a0037507 25244488

[B49] Twamley E. W. Jak A. J. Delis D. C. Bondi M. W. Lohr J. B. (2014). Cognitive symptom management and rehabilitation therapy (cogSMART) for veterans with traumatic brain injury: pilot randomized controlled trial. *J. Rehabil. Res. Dev.* 51 59–70. 10.1682/JRRD.2013.01.0020 24805894

[B50] Vas A. K. Chapman S. B. Cook L. G. Elliott A. C. Keebler M. (2011). Higher-order reasoning training years after traumatic brain injury in adults. *J. Head Trauma Rehabil.* 26 224–239. 10.1097/HTR.0b013e318218dd3d 21552071

[B51] Wai-Kwong Man D. Soong W. Y. L. Tam S. F. Hui-Chan C. W. Y. (2006). Development and evaluation of a pictorial-based analogical problem-solving programme for people with traumatic brain injury. *Brain Inj.* 20 981–990. 10.1080/13561820600909852 17062429

[B52] Wilson M. Zolfaghari P. Griffin C. Lockey D. Tolias C. Verma V. (2014). The future of traumatic brain injury research. *Scand. J. Trauma Resusc. Emerg. Med.* 22(Suppl. 1):A7 10.1186/1757-7241-22-S1-A7

[B53] Wykes T. Huddy V. Cellard C. Mcgurk S. R. Czobor P. (2011). A meta-analysis of cognitive remediation for schizophrenia: methodology and effect sizes. *Am. J. Psychiatry* 168 472–485. 10.1176/appi.ajp.2010.10060855 21406461

