# Supplementary material for: Cognitive Training for Post-Acute Traumatic Brain Injury: A Systematic Review and Meta-Analysis
Source: Front Hum Neurosci. 2016 Oct 27;10:537. doi: 10.3389/fnhum.2016.00537 (PMC5081379; doi:10.3389/fnhum.2016.00537)
Supplement: Supplementary file 2 [file Data_Sheet_1.DOCX]

**Appendix e-2.** Database search strategy for identifying studies of cognitive training interventions for traumatic brain injury.

1. tbi
2. brain damage or brain damaged or brain damages
3. head trauma or head traumas
4. diffuse axonal injury or DAI
5. traumatic
6. acquired
7. intra-cranial or intra cranial or intracranial
8. head
9. brain
10. injur*
11. 5 or 6 or 7 or 8 or 9
12. 10 and 11
13. extra dural or extra-dural or extradural
14. epi-dural or epi dural or epidural
15. sub-dural or 'sub dural' or subdural
16. sub-arachnoid or 'sub arachnoid' or subarachnoid
17. haemorrhage or hemorrhage or contusion
18. 7 or 13 or 14 or 15 or 16
19. 17 and 18
20. 1 or 2 or 3 or 4 or 12 or 19
21. computer training or computerised training or computerized training
22. brain training or memory training or attention training or reasoning training
23. brain exercise or memory exercise or attention exercise or reasoning exercise
24. videogame* or video game or video games or video-game or video-games or computer game or computer games or computer-game or computer-games
25. mnemonic* or method of loci and training
26. speed and processing and training
27. cognitive rehabilitation or cognitive intervention or cognitive interventions or cognitive training or cognitive remediation
28. mental activity or mental activities
29. virtual reality
30. 21 or 22 or 23 or 24 or 25 or 26 or 27 or 28 or 29
31. 20 and 30
